# A qualitative study on the Virtual Emergency Department care experiences of equity-deserving populations

**DOI:** 10.1371/journal.pone.0304618

**Published:** 2024-06-04

**Authors:** Justin N. Hall, Abirami Vijayakumar, Logan Reis, Akm Alamgir, Kristina M. Kokorelias, Julia Hemphill, Noorin Pattni, Laurie Legere, Ilana J. Halperin, Lisa Di Prospero, Debbie Elman, Sharon Domb, Dana Arafeh, Cliff Ledwos, Christine L. Sheppard, Sander L. Hitzig

**Affiliations:** 1 Sunnybrook Health Sciences Centre, Toronto, Ontario, Canada; 2 Temerty Faculty of Medicine, University of Toronto, Toronto, Ontario, Canada; 3 Sunnybrook Research Institute, Toronto, Ontario, Canada; 4 Institute of Health Policy, Management and Evaluation, University of Toronto, Toronto, Ontario, Canada; 5 Access Alliance Multicultural Health and Community Services, Toronto, Ontario, Canada; 6 Sinai Health, University Health Network, Toronto, Ontario, Canada; 7 Wellesley Institute, Toronto, Ontario, Canada; 8 Factor-Inwentash Faculty of Social Work, University of Toronto, Toronto, Ontario, Canada; University of Michigan School of Nursing, UNITED STATES

## Abstract

Patients from equity-deserving populations, such as those who are from racialized communities, the 2SLGBTQI+ community, who are refugees or immigrants, and/or who have a disability, may experience a unique set of challenges accessing virtual models of care. The objective of this qualitative study was to describe the experiences of patients from equity-deserving communities and their family members who received care from a Virtual Emergency Department (ED) in Toronto, Canada. Forty-three participants (36 patients and 7 family caregivers) with different and intersecting identities who used the Virtual ED participated in the study. Semi-structured interviews were conducted to explore reasons for accessing the Virtual ED, barriers to access, and how the Virtual ED met their care needs and expectations, including ways their experience could have been improved. Thematic analysis was used to identify themes from the data. Patients from equity-deserving populations described negative past experiences with ED in-person care, which included recounts of discrimination or culturally insensitive care while waiting to see the ED physician or nurse. Conversely, participants found the Virtual ED to be a socially and culturally safe space since they could now by-pass the waiting room experience. However, virtual care could not replace in-person care for certain issues (e.g., physical exam), and there was a need for greater promotion of the service to specific communities that might benefit from having access to the Virtual ED. Targeted outreach to help raise awareness of the service to equity-deserving communities is an important future direction.

## Introduction

In Canada and beyond, the onset of the COVID-19 pandemic shifted ambulatory care (i.e., outpatient services) to virtual care for both non-urgent and urgent visits [1, 2]. Virtual care has been defined as “any interaction between patients and/or members of their circle of care, occurring remotely, using any forms of communication or information technologies, with the aim of facilitating or maximizing the quality and effectiveness of patient care.” [3]. During the pandemic, the rates of Canadian patients using virtual care services increased from 1.6% in 2019 to 70.6% in 2020 as a result of the lockdown policies that restricted access to in-person care [4]. This included many hospitals enhancing their existing emergency departments (ED) to offer a virtual care option [5]. Through provincial funding, Virtual EDs were established across the province [6] to minimize the need for patients with low risk complaints to seek in-person care as a means to avoid face-to-face contact whenever possible [2, 7].

A Virtual ED that was developed at a major academic tertiary/quaternary care hospital in Toronto, Ontario was launched as a pilot program in 2020. The program enabled patients to book online appointments to have a virtual visit with an ED physician via videoconferencing. Although rapidly designed to decrease the spread of COVID-19, the Virtual ED team examined ways to sustain this new modality of Virtual ED care beyond the pandemic [8]. While some physicians were satisfied with the ability to be able to reach their patients, others were more frustrated by not being able to fully use all of their skills as ED physicians (e.g., physical exam) as well as feeling that not all of their patients’ concerns could be fully resolved because of it [8]. Feeling that virtual care practices were not equivalent to in-person practices, some physicians opted to not provide Virtual ED care [8].

In addition to physician experiences and preferences, the success of virtual care models are also impacted by patient characteristics [9]. For instance, various social determinants of health have been shown to influence access to and use of virtual models of care by people from equity-deserving populations (EDPs) [9, 10]. People from EDPs may include, but are not limited to, the Indigenous community, people from Black or other racialized communities of colour, women and gender diverse people, members of the 2SLGBTQI+ community, people with disabilities, recent immigrants or refugees, and people with low income. Specific examples that contribute to making access and use of virtual models of care by EDPs more difficult may include digital inequities (including low digital literacy and lack of access to Wi-Fi or smartphones/laptops, etc.), language barriers, and no private space to have a confidential appointment [10–12].

In addition to matters of digital equity that can affect access to virtual options, there remain questions regarding the quality of care that people from EDPs may receive from the Virtual ED [8, 13]. Across diverse groups, there are documented reports of prejudice for EDPs for in-person ED visits, which include disrespect, neglect and/or overt racism directed towards the Indigenous community, persons with mental health issues, and the transgender community [14–16]. As a result, there is not only a need to explore barriers to virtual urgent care for EDPs but also a need to learn from people from diverse EDP communities on their perceived quality of the care experience to improve virtual care programs.

The main objective of this qualitative study was to describe the experiences of patients from EDPs and their family members who received care from a Virtual ED. Specifically, we were interested in how virtual care options compared to in-person care and what features of virtual care promoted or hindered equitable access and experiences that might be commonly experienced across patients of diverse backgrounds. By examining these issues, a clearer understanding of EDP preferences for Virtual ED can be obtained, and then mobilized into a set of best practices to optimize equitable and compassionate virtual care. Importantly, in the post-pandemic landscape, virtual models of care may serve as a means to enhance access to care and service efficiency [17], which can help fill essential gaps in care currently being experienced by the Canadian healthcare system [18]. Given the expressed interest in maintaining Virtual EDs in the long-term [8], this is a critical time to seize the opportunity to engage with patients about models of care and associated technologies being used to determine how they can be best used to support high quality care [19].

## Material and methods

An inductive qualitative study [20] was used to examine the experiences of patients from EDPs who used the Virtual ED. A qualitative approach is especially useful for exploring the experiences of Virtual ED care by EDPs since there are limited studies around Virtual ED care [8], and the concepts of interest (i.e., EDPs). The study received ethics approval from the Sunnybrook Health Sciences Centre’s Research Ethics Board (REB #5080).

### Virtual ED

The Virtual ED initiative under study commenced in December 2020 during the COVID-19 pandemic, and was a partnered initiative across three major urban hospitals in Toronto, Ontario [8, 21]. The Virtual ED was designed to provide people the ability to schedule a same-day appointment with any of the partner hospitals through the ED’s online platform and to choose an appointment time that is most convenient for them. The service is intended for Torontonians who have an urgent medical issue that is not life threatening (e.g. body aches, rashes, nausea, etc.), and who are unable to make an appointment with a family doctor or nurse practitioner. The Virtual ED operates daily and was staffed by a single ED physician at a time from a roster of 22 physicians. The Virtual ED was promoted by advertising it on social media (Facebook, Instagram, LinkedIn, Twitter), through printed posters at community health centres and family practice offices, posters and pamphlets in the in-person EDs, through digital newsletters shared by various community and social support organizations, and in various community service directories.

A dedicated patient administrative associate confirms demographic details that have been entered by the patient using the online platform, validates their heath card, and creates an appointment that is emailed and/or texted to the patient. All appointments are completed via Zoom (Zoom Video Connections) with phone as a back-up option. At the end of the Virtual ED visit, one of five outcomes is possible: (1) a resolved issue during the visit with no follow-up required, (2) a recommended follow-up with family physician, (3) a referral to a specialist for follow-up, (4) a scheduled follow-up with diagnostic imaging and/or laboratory testing, or (5) a recommendation to attend the in-person ED for further assessment and management. Documentation is completed within the electronic health record system, which links to the provincial Health Report Manager (HRM) and Connecting Ontario to ensure shared access by providers outside of the organization.

Between the program inception and September 2023, the Virtual ED saw over 5,800 patients, with 65% of those being female, and 81% indicated they had a family physician. In terms of age ranges, less than 5% were younger than 18 years old, 37% were between the ages of 18–34 years, 30% were 35–50 years, 15% were between the ages of 51–65 years, 9% were between 65–79 years, and 4% were 80 years and greater. Reasons for visiting the Virtual ED were most commonly linked to orthopedic, skin and soft tissue related (e.g., rashes), gastrointestinal, infectious or system navigation concerns. Eighty-five percent of patients were those that were well-managed via the Virtual ED visit (did not require in-person ED assessment).

### Participants

A heterogeneous purposive sampling technique with an intentional selection of diverse participants was used to recruit the sample [22]. Maximal Variation Sampling was used to include diverse participants with key attributes of variation so that the sample would be representative of the diverse population of interest [23]. The inclusion criteria were adult (18 years or older) patients and/or their family members who had used the Virtual ED within the last 2 months of study contact. Any patient who self-identified as a member of an EDP was eligible to participate. This included people who are Indigenous, Black or racialized, people with disabilities, persons from the 2SLGBTQI+ community, people with limited English proficiency, recent immigrants or refugees to Canada, people with a low-income or anyone else who felt they may face a disadvantage in accessing and receiving care. There were no language restrictions as our research team had access to a certified translation service.

### Data collection

Recruitment and data collection for the study occurred between March 21, 2022 and September 7, 2022. All Virtual ED patients who were patients of the Virtual ED during this period were shared a link to an information letter in English to learn about the study and an online pre-screening tool to indicate if they were interested in learning more. This included an option for non-English speaking participants to receive information in their preferred language via a translator. The pre-screening survey collected data on EDP identity, which included a set of categories a person could select from to indicate their identity(ies). If the list of pre-defined categories did not capture their EDP identity, the participant could enter a description in a text box on the form. If persons self-identified as being from an EDP, they were asked to provide their contact information or to contact the study staff.

Family members who accompanied the patient during the Virtual ED visit were permitted to be an interviewee with the patient if they both desired to do so. We included family members in the study because the populations we were interested in are those that often face challenges with the healthcare system due to barriers in English language proficiency, being a newcomer, or requiring additional supports due to their disability. Consequently, patients often bring a family member to help them navigate these issues for in-person appointments, which was not always possible during the pandemic. With this in mind, we felt it important to include family members in the study to provide additional context and insights on how they viewed the treatment of their family members by the Virtual ED compared to previous in-person visits.

A total of 1,150 invites were sent to people who had used the Virtual ED during this time period. One hundred and sixty-two respondents completed the online eligibility screening tool, with 89 self-identifying as belonging to an EDP (see Table 1). Of those, 73 agreed to be contacted about the study by providing their phone number and/or email address. The research staff then contacted the potential participants by phone or Zoom to screen for eligibility, and to obtain informed verbal consent.

**Table 1:** Screening survey for EDP identities (N = 89).

A Community of Colour	47
People with Disabilities	21
Persons from the 2SLGBTQ+ Community	17
People who prefer to communicate in languages other than English	11
Recent Immigrant to Canada	10
People with a Low-income	19
Prefer not to disclose	3
Other–not described	6

Note: Some respondents indicated that they belonged to more than one equity-deserving community.

Verbal consent was documented for participation into the interviews using a form indicating that the research staff explained all the relevant details of the project understandably, including voluntary option to participate and withdraw without prejudice, risks and benefits, and issues of confidentiality and anonymity. The completed verbal consent forms were sent to the participants for their records. The interviews were then scheduled shortly thereafter. Two participants did not meet the eligibility criteria while others did not respond to follow-ups or indicated they were not interested after learning more about the study. Thirty-six patients consented to take part in the interviews with 4 preferring to do it in their native tongue (2 in Mandarin, 1 in Spanish and 1 in Farsi). Some participants also chose to undergo the interview with their family member (n = 7). Hence, the total sample was 43 participants.

After consent was obtained, data were collected by two trained cisgender White women (both with masters degrees), and one cisgender South Asian man who was a foreign trained public health physician (also with a masters degree) who were working as research staff on the project. The interviews with participants explored what influenced them to access the Virtual ED, the barriers they faced accessing it, whether the Virtual ED met their care needs and expectations and/or how their experience could have been improved. See S1 File for the interview guide. All the family members (n = 7) who participated underwent the interview with their family member who sought care from the Virtual ED. All the interviews were conducted via a secured Zoom account, and lasted approximately 1 hour. None of the interviewers had ties to the Virtual ED nor had a prior relationship with the study participants. After the interview was completed, participants were thanked and provided a $25 gift card. Interviews were professionally audio-transcribed verbatim.

### Data analysis

The data were analyzed using Codebook Thematic Analysis (TA) as described by Braun and Clarke [24]. This approach is characterized by the use of a coding framework, and the belief that data need not be “accurately” summarized, but rather, reflexively and subjectively interpreted by researchers [24]. Although not used to facilitate interrater agreement, codebook TA can be pragmatically used to facilitate the process of having multiple data analysts code a dataset [24]. Codebook approaches recognize the interpretive nature of data coding and conceptualizes themes as domain summaries [25].

The data analysis commenced during the data collection period. Members of the team with training in qualitative methodologies (SLH, JH, NP) reviewed the transcripts to establish a codebook. Once established, two research staff (JH and NP) independently coded a sub-set of the transcripts until they reached inter-coder agreement, whereby sections of text were being coded similarly or points of disagreement were easily resolved. The codebook used to categorize the data was then reviewed by the members of the investigation team (JNH, KMK, CLS, SLH) with qualitative research and/or content expertise (i.e., Virtual ED care, health equity).

During the course of the analysis, the two staff who initially established the coding framework left to pursue new professional opportunities. This resulted in having another two staff with formal training in qualitative methodologies (LR and AV) take over the coding process, who consulted with the previous staff (JH and NP) about the codebook. The two new staff (LR and AV) then continued the analysis process by independently coding the transcripts until a high level of inter-rater agreement was achieved. This process was overseen by the senior responsible author who worked to establish the initial codebook (SLH). All data were coded using NVIVO 12 software to organize the study data [26].

Regular peer debriefing meetings were held between the two coders (LR and AV) with the senior-responsible author (SLH) to review the identified themes to determine which ones needed to be refined, broadened, or eliminated in light of how readily they adhered to the data set. Lastly, themes were finalized, named and defined with the authors engaged in the analytical process (LR, AV, JNH, SLH), and a detailed audit trail was finalized to document the criteria by which the data fit each theme.

Through team discussion, we determined the study had sufficient information power [27]. The determination of information power was based on the alignment of the study’s aims with its exploratory nature, focusing specifically on EDP experiences with a Virtual ED. Additionally, the richness and depth of information derived from the interviews, allowing for the discernment of common themes across diverse EDP groups, contributed significantly to the assessment of sufficient information power.

## Results and discussion

The study team interviewed 36 patients and 7 family caregivers with intersecting identities (see Table 2). Most of them self-identified as women, (n = 35), with the rest being men (n = 6) or non-binary (n = 2). Most were heterosexual (n = 34) and 9 identified as gay, bisexual or queer. Twenty-two were from communities of colour and most had strong levels of English and digital fluency. In terms of health status, many had a chronic illness (n = 22) or some type of disability (n = 21), and 16 reported having a mental illness. Some of the reasons noted for visiting the Virtual ED included having some type of chest or abdominal pain, an injury (e.g., hitting head), breathing difficulties, a flare-up of symptoms from a pre-existing condition (e.g., asthma), or experiencing other symptoms such as sore throat, vomiting and fever (see Table 2).

**Table 2:** Demographic information of participants (N = 43).

Characteristic	Patient (n = 36)	Caregiver (n = 7)
	n, %	n, %
**Born in Canada**		
Yes	19 (53)	5 (71)
No	17 (47)	2 (29)
**Gender**		
Women	28 (78)	7 (100)
Men	6 (17)	
Non-Binary	2 (5)	
**Sexuality**		
Straight	27 (75)	7 (100)
Gay	5 (14)	
Bisexual /Queer	4 (11)	
**Age**		
18–25	5 (14)	0 (0)
26–35	10 (28)	1 (14)
36–45	11 (31)	2 (29)
46–55	5 (14)	2 (14)
56–65	3 (8)	1 (14)
66–75	1 (3)	1 (14)
76+	1 (3)	
**Ethnicity**		
Black	1 (3)	
East Asian	6 (17)	1 (14)
Latin American	2 (5)	1 (14)
Middle Eastern	2 (5)	
South Asian	10 (28)	1 (14)
White	11 (31)	4 (57)
Jewish	3 (8)	
Did not disclose	1 (3)	
**English Proficiency**		
Poor	2 (5)	
Fair	1 (3)	
Good	3 (8)	1 (14)
Very Good	6 (17)	2 (29)
Excellent	24 (67)	4 (57)
**Technology Proficiency**		
Poor	1 (3)	
Fair	1 (3)	
Good	4 (14)	2 (17)
Very Good	11 (29)	4 (57)
Excellent	19 (53)	1 (14)
**Health Factors**		
Chronic Illness	22 (62)	4 (57)
Developmental Disability	1 (3)	
Sensory Disability	1 (3)	
Drug/Alcohol Dependency	2 (6)	
Learning Disability	9 (25)	
Mental Illness	16 (45)	1 (14)
Physical Disability	10 (28)	1 (14)
None	9 (25)	2 (29)
Prefers not to say	1 (3)	
**Total Family Annual Income**		
$0-$14,999	4 (12)	
$15,000-$19,999	2 (6)	
$30,000-$34,000	1(3)	1
$40, 0000-$60, 000	1 (3)	1 (14)
$60,000+	25 (69)	4 (57)
Did not disclose	3 (83)	1 (14)

From the analysis, three main themes were identified: 1) inequities experienced by EDPs receiving in-person care; 2) ways Virtual ED promoted safety; and 3) opportunities to enhance Virtual ED care for EDPs. The first theme recounts the negative experiences patients encountered when reflecting on past in-person care, which was discriminatory or culturally insensitive. The second theme describes how the virtual experience was seen as being “safer” since it afforded participants more control over the care experience. Finally, the last theme details the broad benefits and challenges associated with the Virtual ED, as well as specific suggestions on how to enhance the virtual care experience. Although our sample was diverse, and had intersecting identities (gender, sexual orientation, racial/ethnic background, etc.), the majority often discussed only one aspect of identity in how it influenced their care experiences (or that of their family member). The themes are described below with sample quotations to help illustrate the themes.

### Theme 1: Inequities experienced by EDPs receiving in-person ED care

Negative past experiences with in-person care were common among participants, with many experiencing at worst some form of prejudice or discrimination and at best, insensitive or non-compassionate care. One participant noted:

“*Even when I went to the in-person hospital*, *they were like*, ***is there any chance you could be pregnant? And I said*, *no*, *my partner is a trans man***. *And they’re like*, *oh*, *so they’ve already had their surgery so there’s no sperm? I was like*, *no*, *they’re a trans man so there was never any sperm to begin with*. *And I have to go into this whole thing*, ***and they still did a pregnancy test*. *And I’m like*, *why*, *what’s the point?***”

*(ID#57*, *Non-Binary Person*, *18–25 years*, *Pansexual*, *White*, *Physical Disability & Several Other Conditions)*.

In some cases, people felt that their status as part of a visible minority affected their medical care, with one person with a history of asthma stating: *“I feel like I am always taken for granted that I am not at the highest seriousness when I’m clearly experiencing an [asthma] attack*.” *(ID#27*, *Bisexual Woman*, *South Asian*, *Chronic Condition*, *Physical Disability & Several Other Conditions)*. Other forms of discrimination arose in part because of the lack of accessibility of the physical space, with one family caregiver discussing how they felt their family member with a disability had to wait unnecessarily in the waiting room during a past in-person visit because appropriate accommodations were not available. As a result, the family member felt the situation had become discriminatory.

“*… And I said*, *well*, *she did wait her turn*, *and her turn came up*, *and actually her turn’s come up twice now*, *and I understand that her situation creates some difficulties and I get that and that’s why we’ve continued to wait*, ***but now it’s flipping into discrimination***. *Now she’s waiting longer because she has a disability*, *as opposed to […] You’ve had two hours now to figure out the accommodation*.”

*(ID#FC1*, *Heterosexual Woman*, *46–55 years*, *White European*, *Family Caregiver)*.

Others noted they would need to monitor how they presented themselves to healthcare providers in order to avoid discrimination, with one participant recalling:

*“…I think for me*, *I can’t say that I haven’t felt that certain experiences have been* ***because nobody wants a vocal brown girl*…*I’m saying this because this is the struggle of every person of colour***. *You think*, *was it really because of that*, *should I say something*, *should I not say something*, *and you just start to question everything*. *I’m sure these are not things that you [Referring to JH Interviewer] have to think about… whereas I’m like*, *if I sound too overbearing*, *chances are I’m going to be again put at the bottom of a…There’s a lot of that that happens in general on a daily basis in every aspect*, *so a visit to a doctor or an ER is no different*, *where you’re like*, *just be nice*, *just smile*. *Actually*, *as I’m talking to you*, *I just* ***realize I almost go out of way*. *I’m extra nice*, *put on a smile*, *and approach with caution****”*.

*(ID#66*, *Heterosexual Woman*, *46–55 years*, *South Asian*, *Chronic Illness)*.

Interestingly, many participants felt that many of the instances of inequitable care did not stem from their interactions with the physicians or nurses providing direct care but rather more with staff working in the ED (e.g., administrative support workers). For instance, patients reported ED staff being unaccommodating to patient’s physical limitations before even seeing a physician. One participant recounted how they were denied the ability to sit where they would prefer:

*“I have*, *in the past*, *been at the ER and said that I need to sit outside*, *and if I’m going to be here for an hour can I do that*, *and you can come and get me?* ***Because it’s not comfortable for me to just stand there for two hours and sitting and standing causes me pain*. *And they’ve always said no***.*”*

*(ID#29*, *Heterosexual Woman*, *30–45 years*, *Ashkenazi Jewish*, *Low Income*, *Chronic Condition & Physical Disability)*.

Furthermore, patients who did not have a provincial health care plan (i.e., Ontario Health Insurance Plan [OHIP]) recounted how their lack of OHIP eligibility made them feel “lesser”; as if they were being triaged differently due to perceived discrimination. For example, one participant expressed:

*“****Yes*. *I felt that I was less***, *they will give me less treatment in terms of the… I mean less time on the treatments because I don’t have the OHIP*. *I don’t pay for it*, *I don’t pay for the hospital […]I can’t say*, *but treatment in the emergency room*, *I felt that I didn’t get that much*.

*(ID#1*, *Heterosexual Woman*, *46–55 years*, *South East Asian*, *Chronic Illness)*.

Overall, participants discussed several instances of how in-person care led to situations that were inequitable, which they felt were a result of their EDP status.

### Theme 2: Ways Virtual ED promoted safety

The Virtual ED made participants feel safer in accessing care, as it avoided some of the triggering issues associated with receiving in-person care. For instance, having access to the Virtual ED reduced stress for participants, with one person reporting that it enabled them to bypass the whole experience of sitting in a waiting room with other patients: “***I felt safer doing it virtually than I would going in*, *especially with my mental health***. *It can be negatively affected by being in hospitals*.” *(ID#4*, *Bisexual Woman*, *18–25 years*, *White*, *Low Income*, *Chronic Condition*, *Physical Disability & Several Other Conditions)*. This ability to bypass other patients as well as other staff who they experienced negative interactions with (i.e., prejudice, anxiety) provided a greater sense of control over their care. As well, participants talked about how the Virtual ED enabled them to circumvent COVID-19 restrictions of having a family member be present during their care. One participant stated: *“****The virtual part of it is I can have anyone [family caregivers] around me*** *… I don’t have to worry that there will be rules against it*.” *(ID#12*, *Heterosexual Woman*, *18–25 years*, *White*, *Chronic Illness*, *Learning Disability*, *Mental Illness)*.

The problems raised with in-person care appeared to be minimized when participants were commenting on the quality of the care interaction, with one person saying: “*They did not cut me off when I was speaking*. *They did not say that I was incorrect for how I was feeling*, ***so I was not invalidated in any way***.” *(ID#27*, *Bisexual Woman*, *26–35 years*, *South Asian*, *Chronic Condition*, *Physical Disability & Several Other Conditions)*.

While having a provincial health card (i.e., Ontario Health Insurance Plan) is not required to use the service, a few persons in our sample without OHIP cards expressed an added sense of safety with the accessibility of the Virtual ED service: *“****I felt a relief****…I have this feeling that someone will ask for the OHIP or my eligibility or there will be someone who will ask for how will I… That’s always my thinking*, *how am I going to pay for this consultation*?*” (ID#1*, *Heterosexual Woman*, *46–55 years*, *South East Asian*, *Chronic Illness)*. Overall, there were many advantages to using the Virtual ED over in-person visits, which created a more empowering care experience for patients across EDP memberships.

### Theme 3: Opportunities to enhance Virtual ED care for EDPs

Across all the interviews, it was apparent that the participants had many positive reflections about their Virtual ED experience compared to previous in-person visits, with many of the benefits being widely applicable to a variety of patients (e.g., avoiding costs and saving time with not having to travel to the hospital, less waiting time, etc.). Many participants discussed the ease, convenience and flexibility of the Virtual ED, including the use of the online booking system, and commented liking that they did not have to travel to the ED or wait for long times to see the physician. One person stated: “*It was easy*. *I know how to use technology*, *so it’s pretty easy*. *And the nurse*, *she was professional*.” *(ID#19*, *Heterosexual Man*, *18–25 years*, *Black)*.

Similar to the benefits, there were noted challenges that were likely universal. For instance, the Virtual ED was limiting due to the inherent features of using digital technologies as a care modality. The inability to physically assess all patient concerns was a disadvantage, and participants felt that virtual care could not replace in-person care for everything:

*“But I think in-person is obviously better because you’re actually face to face with the doctor*. *Maybe she can do some checks*, *stuff like that*. ***So when it comes to easier access*, *I think an online one is better*, *but when it comes to effectiveness I guess*, *I would say obviously an in-person one is probably better***.*”*

*(#19*, *Heterosexual Man*, *18–25 years*, *Black)*.

The technology itself also created barriers for some. For instance, the online booking system was a source of frustration for those whose health conditions required additional accommodations to make the digital platform more accessible. One person who had a learning disability explained: *“One of the things is that when you’re filling out the application*, ***there is a time limit as to how long you have to have it done in…and actually*, *one of my learning disabilities makes that complicated***.*” (ID#4*, *Bisexual Woman*, *18–25 years*, *White*, *Low Income*, *Chronic Condition*, *Physical Disability & Several Other Conditions)*. These types of challenges with navigating the booking system and Virtual ED thought to be potentially more difficult for patients from certain equity-deserving groups, such as older people or those with sensory impairments.

*“* ***If I was older*, *maybe my father*, *he would probably not know what to do if that was him*, *and probably never even access it*.*”***

*(ID#33, Heterosexual Woman, 36–45 years, South East Asian, Chronic Illness)*.

“..***I wasn’t sure how accessible it would be to [relatives who have visual or auditory impairments]*** …*I couldn’t find where it was advertised that it would be for people who are, for example, blind or people who are deaf. I couldn’t see the signup process, how that would have worked for someone who is in that situation*.”

*(ID#4*, *Bisexual Woman*, *18–25 years*, *Low Income*, *Chronic Condition*, *Physical Disability & Several Other Conditions)*.

Others experienced challenges because of the type of device they were using to access the service, with some stating that the Virtual ED was better suited for use on a larger device (e.g., computer). One person who lacked a computer stated:

*“****Because having a computer with internet access and Zoom installed and the ability to use Zoom is quite a lot to ask for large sections of the population***, *but phone is pretty low barrier*. *Most people do have access to a phone and I think that that’s something that would broaden accessibility of this service to people that it would be really helpful for*.*”*

*(ID#45*, *Homosexual Male*, *36–45 years*, *White*, *Previous Mental Illness)*.

One useful support offered by the Virtual ED that helped address some of these issues though was the ability to receive assistance from the administrative staff who helped some of the participants to fill out the application and to set up the appointments.

*“Something that actually was really nice was someone* ***called me about half an hour before my appointment to double check that I had received the Zoom link and was ready to be contacted***. *Actually*, *I was okay because I use Zoom all the time so I’m very comfortable with it*, *but I think for someone who maybe isn’t as comfortable with Zoom or telehealth*, ***it was really nice to have someone check in on you to make sure that you are ready for the appointment***.*”*

*(ID#37*, *Heterosexual Woman*, *26–35*, *White*, *Learning Disability)*.

Overall, the technology-dependent limiting factors to using and accessing the Virtual ED were deemed inconvenient at most, with the hope that possible recommendations for improvement will be taken seriously to improve the service.

Throughout discussions with EDPs and their family members, several suggestions on how to best optimize Virtual ED for EDPs emerged. To promote and create a more inclusive virtual care experience, some participants talked about having staff display their pronouns and/or have other background imagery reinforcing the safety of receiving care in the Virtual ED. One suggested:

*“But I think it would be* ***a good idea for some people who are more sensitive to this issue to have the preferred pronouns***. *So that could be like a very good idea [for the physician to display their pronouns in Zoom]*.*”*

*(ID#14*, *Gay Man*, *36–45 years*, *Latino*, *Chronic Illness*, *Mental Illness & Learning Disability)*.

Other suggestions to promote inclusivity in a virtual care setting included using nametags and providing options on how one could self-report their EDP identities:

“***on peoples’ name tags*, *like in peoples’ tags*, *right*, *because you’ve got the ability to have something*** *that would be… So around Pride and stuff at my hospital we have [Pride] pins*, *and we start wearing them around our nametags*. *But yes*, *almost to just be intentional in the creating of the space*. *It’s similar to how people create safer spaces in their offices*”

*(ID#2, Homosexual Female, 36–45 years, White, Chronic Illness)*.

“*Sometimes when you select other there’s an* ***option to self-identify***, *which there isn’t in this case*. ***I think that might be helpful*. *It’s more respectful*, *it helps people feel like their identity is captured and that it matters***. *And a lot of people don’t like being*, *literally*, *othered*. *So*, *firstly*, *having the term*, *other identity*, *I think is more respectful than other*. *And then having a dropdown option so that people can self-identify ensures that people are seen and counted*”

*(ID#43*, *Non-Binary Person*, *26–35 years*, *White*, *Chronic Illness*, *Mental Illness*, *Temporary Disability)*.

In terms of making the service more accessible, people discussed the need for more language supports for the booking portal and during appointments for non-English speaking individuals. For instance, one person noted: *“I’ll be honest with you*, *if the person*, ***if someone can’t sign up for that*, *it would either be they don’t understand English or they can’t read or write or something in English***.*” (#19*, *Heterosexual Man*, *18–25 years*, *Black)*. As a result of these limitations, people with low levels of English fluency may have chosen to attend the in-person ED.

*“As for my mum*, *she had to use the emergency services as well*, *but* ***she couldn’t do this online because right now there is no Chinese language version available***, *so often she had to actually come to the emergency department in person*. *Have you used this online?”*

*(ID#15*, *Heterosexual Woman*, *36–45 years*, *Chinese–East Asian)*.

Some participants spoke to the notion of equity, and how providing a service with access to multiple languages could increase its reach and ability to attend to a wider range of health concerns. One person noted: “*The doctor was very polite and very good*, *but I would have felt better if I could express everything*, *because I have limited English understanding*, *if I could have done that in Spanish” (ID#10*, *Heterosexual Woman*, *46–55 years*, *Latin*, *Mixed*, *Chronic Illness*, *Mental Illness)*. As well, another person discussed that language barriers might hinder the ability to obtain a rich description of the person’s concerns, which could be tied to their ethnicity:

*“That might be beneficial*. *And then*, *thinking also in terms of equity*, *I think language might be one of them*. ***And I guess this would depend on the type of condition that the patient is seeking care for*, *but I think being South Asian is a risk factor for certain conditions***.*”*

*(ID#13*, *Heterosexual Woman*, *26–35 years*, *South Asian)*.

Participants also noted the need to create greater awareness of the Virtual ED service, and that this outreach should be targeted to specific EDP communities since many individuals are unaware of its existence:

“*But also I don’t know how many people actually know this option*. ***There are ways on the Internet to promote certain things*. *If there’s a way to promote spreading this information*** *that this option exists for care*, *I think that would be useful as well*. *Again*, *I really don’t think that many people know about it*”

*(ID#42*, *Heterosexual Woman*, *26–35*, *South Asian)*.

A greater awareness of the Virtual ED would also provide EDPs the opportunity to circumvent some the challenges with in-person care (detailed in Theme 1 above). For instance, participants from the 2SLGBTQI+ community who took part in the study expressed concerns about potential discrimination and receiving inadequate care if they were to visit an in-person ED.

“***I feel like you guys should actually advertise more in our [2SLGBTQI+] community*** *…I saw it at my work*. *I live in the village…*. *Targeting the community… Especially with the monkeypox going on*. *I have some friends who caught it and then they have symptoms that are COVID symptoms*. *Who’s going to really check you?*”

*(ID #64*, *Gay Male*, *26–35*, *South East Asian)*.

This highlights the need to raise awareness about the Virtual ED service as a safe and inclusive healthcare option for individuals across EDP communities.

## Discussion

The main objective of this qualitative study was to describe the experiences of patients from diverse EDPs who received care from a Virtual ED and that of their family members. Specifically, we were interested in how virtual care options compared to in-person care and what features of virtual care promoted or hindered equitable care for a variety of persons from diverse backgrounds. As a point of comparison in discussing the advantages and disadvantages of the Virtual ED, many of our participants drew upon their past negative in-person ED experiences to help illustrate their points. These experiences align with other reports in the literature about discriminatory practices in ED care [28], which leads to sub-optimal care and patients feeling unsafe as a result [29–31]. For example, there is evidence that people from the Black community feel their race contributes to them being disrespected by providers and sub-optimal care compared to patients who are White [29]. As well, there is evidence that patients who are Black or Hispanic tend to be assigned lower-acuity triage scores than patients who are White, even though the chief symptoms are identical across groups [30, 31]. Hence, our findings expand on these studies [29–31] as similar experiences were reported by patients from other EDP populations (i.e., South Asian community).

Conversely, participants across EDP groups discussed how they felt safer and in more control of their care when using the Virtual ED. These findings are consistent with previous studies that describe how virtual care is viewed as a culturally and personally safe modality [32–34]. One qualitative study exploring Indigenous experiences identified safety as a theme, where participants felt in control of their care since they had the option of selecting either a male or female doctor, which led to increased autonomy [32]. In our study, the ability to bypass the waiting room experience was a major factor that promoted feelings of safety, since most of our sample’s past negative interactions were dealing with the initial process of registering to see a physician or nurse or while sitting in the waiting room. As such, there are several features of the Virtual ED that appear to lend itself to being a more inclusive and culturally safe environment for EDPs to receive care.

Similar to other reports on virtual care [8, 35], there were challenges accessing and navigating technology due to language barriers or disability (e.g., learning disability, sensory impairments, etc.). Importantly, participants across EDP groups discussed the need for targeted outreach to help raise awareness of the service to their community. While Virtual ED care has grown rapidly, it remains in its infancy, and our work critically identifies the importance of supporting multi-modal communication to increase awareness amongst individuals and communities that may most benefit from this type of service. This includes those who do not have access to a primary care longitudinal provider (family physician or nurse practitioner) and those who belong to EDPs. The engagement of community organizations providing services to under-served groups may help to achieve this goal and can also provide important insight into how to best deliver care in a compassionate and culturally competent way [36].

As part of a comprehensive communications and outreach program, there is a need to not only increase awareness but to also share information on what patients can expect in terms of care in various formats (e.g., in-person versus virtual). As described by our participants, many felt there was a need to raise a higher level of awareness to specific communities who might benefit from the Virtual ED (i.e., 2SLGBTQI+). To effectively share this information and reach EDPs, a multi-pronged strategy is likely to be most effective and include materials such as posters at social, community, and health care locations where EDPs attend, social and traditional media advertisements, websites, and newspaper/magazine advertisements, using language and graphics that are accessible and available in multiple languages [37]. The important role of the administrative personnel in supporting service navigation and follow-up was also viewed as an equity-supporting strength by our participants (e.g., follow-up call), and represents an opportunity to further expand this role to enhance the patient experience.

Equally important is communicating that this is an urgent service for acute medical conditions and helps serve as a bridge, rather than as a replacement for a longitudinal provider. Relatedly, it is a service that was not designed for a particular EDP but rather a widely available public service. As such, providing resources for patients to secure a family physician via Health Care Connect (Ontario) [38] or local family health practices and/or specialist referrals will better support continuity of care [39] and patients’ long-term health. As noted in our data, the advantage of the Virtual ED may be how it is perceived as a “safer” space for EDPs, which could serve as a critical pathway towards more equitable care by helping people navigate to appropriate services for follow-up.

While the current study examined the perspectives of EDPs, a key next step involves interviewing Virtual ED providers to gain their perspectives about providing care to EDPs. Integrating these perspectives and creating a partnership amongst patients, providers, communities, and policymakers will foster autonomy, collaboration, and co-design of improved Virtual ED care services, which is currently being pursued by members of our investigation team. All these key interest groups must be engaged in the continued iterative design, delivery, and evaluation of Virtual ED care services to ensure an equitable, integrated, and sustainable system. Moreover, given the limited training opportunities for established and in-training providers [40], co-creating training opportunities for providers through the lived experience of EDPs will help ensure capacity building in health equity and cultural safety. Importantly, the end of the pandemic has led to discussions whether Virtual EDs should be sustained or if resources should be re-directed back to in-person care. However, Canada is facing an ED crisis, which includes over-crowding [41]. Hence, Virtual EDs may provide one mechanism for minimizing these issues, and to optimize care experiences for persons from EPDs with urgent but non-life threatening conditions. In total, this will allow for the generation of a set of priorities, policies, and Virtual ED best practices for EDPs that can be shared provincially and beyond, and may have implications for other types of non-urgent virtual care.

## Limitations

We sampled from a group of patients during the COVID-19 pandemic who all were able to access the Virtual ED. As such, we are missing the perspectives of EDPs who may have tried to access the Virtual ED but were unable to for a variety of reasons (e.g., low digital or English literacy to navigate the online booking system, lack of a private or safe space to access, etc.). Although our study sample was quite diverse with many having intersecting identities, this may minimize some of our insights on the Virtual ED care of particular groups (e.g., 2SLGBTQI+ individuals, people with disabilities, communities of colour, etc.). However, our goal for this paper was to draw some common and unifying considerations around providing Virtual ED to a variety of EDPs. There are several studies on specific EDPs that highlight the common benefits (e.g., safe) and challenges (e.g., discrimination) associated with in-person and virtual care. Hence, these insights can be mobilized into general practices that can serve to make a widely available service more equitable in its delivery to diverse populations who may wish to access it for their care. Future work will aim to focus more specifically on the care experiences of particular EDPs. As well, future studies should consider different theories of access [42] to help better elucidate different factors that hinder or facilitate EDP access to virtual models of care.

## Conclusions

The participants in our study from EDPs preferred virtual emergency healthcare under certain circumstances as they found the Virtual ED to be a more socially and culturally safe space with improved access compared to their experiences of in-person ED. There remain opportunities to enhance practices and the technology itself for EDPs. As well, targeted outreach to help raise awareness of the service to EDP communities is an important future direction.

## Supporting information

S1 File Interview guide. (DOCX)
