# Supplementary material for: A qualitative study on the Virtual Emergency Department care experiences of equity-deserving populations
Source: PLoS One. 2024 Jun 4;19(6):e0304618. doi: 10.1371/journal.pone.0304618 (PMC11149852; doi:10.1371/journal.pone.0304618)
Supplement: S1 File — (DOCX) [file pone.0304618.s001.docx]

**S1 – INTERVIEW GUIDE QUESTIONS**

1. Please tell me a bit about yourself and why you (or your family member) accessed care in the virtual emergency department.
   1. *How did you first hear about the virtual department?*
   2. *What brought you (your family member) to the virtual emergency department?*
2. Walk me through the process of accessing the virtual emergency department.
   1. *How easy did you find the system to navigate? How easy was it to book an appointment?*
   2. *What help or support did you need accessing the clinic?*
   3. *What kinds of things made it easy for you to access the virtual clinic?*
   4. *Does anything else stand out?*
   5. *When thinking about your experience, was there anything about who you were that may have affected your experience? For instance, was there anything about your language (e.g., ability to speak English), cultural background, your race, gender or sexual orientation?*
3. Please tell me about your experiences with the virtual emergency department hospital staff.
   1. *What kinds of supports or information did they provide (to you and your family member)?*
   2. *What help or support did you not receive but wish you had received? (Information? Emotional support? Somebody doing something for you? How did you receive that help?)*
   3. *How would you explain quality of the care you received? Was it compassionate? Why/why not? Can you give any examples of compassionate care?*
4. What was it about the virtual emergency department that helped you (and your family member) the most?
   1. *What did you (or your family member) find useful about having access to virtual care?*
   2. *Was there any thing that did not help you? Why do you think these were not helpful?*
   3. *What aspects of your personality or life experiences do you think influenced your experience with the virtual urgent care clinic? How did this influence your need for support? Again, this may also be related to your language (e.g., ability to speak English), cultural background, your race, gender or sexual orientation?*
5. When reflecting on your experience of receiving care at the virtual emergency department, would you say it was a good experience or a bad one?
   1. *If a* good experience*, what made the experience a positive one? What made you feel heard and respected?*
   2. *If a bad experience, were there any experiences where you felt that providers ignored you or offended you because of (your disability, skin colour, English-language abilities or anything else about who you were)?*
   3. *Did you feel that the virtual care staff understood your healthcare needs as a member of (an under-served group)? If so, why?*
   4. *Have you ever experienced discrimination in the emergency department, in-person? When we speak of discrimination, we mean unfair treatment of people based on categories such as race, age, or gender.*
   5. *Are there any other challenges you face in accessing care because of your (group)?*
6. If you have had previous experience with in person emergency departments, how did the care provided by the virtual emergency department compare?
7. What parts about the virtual emergency department do you think could be improved upon? What other things could the hospital providers have done differently to make your virtual visit more compassionate?
   1. *Were there things you were hoping the healthcare providers could do for you but didn’t?*
   2. *What advice would you give to other patients/family members considering using virtual care?*
   3. *What advice would you give to the hospital to help improve their virtual care program?*
8. Were there any other topics you expected to discuss that we did not cover today? Do you have any other comments?
   1. *Is there anything else about your time with the virtual emergency department clinic that you would like to share that I didn’t ask you about?*
